# Unravelling interrelations between chemical composition and refractive index dispersion of infrared-transmitting chalcogenide glasses

**DOI:** 10.1038/s41598-018-33824-x

**Published:** 2018-10-19

**Authors:** Jun Ho Lee, Ju Hyeon Choi, Jeong Han Yi, Woo Hyung Lee, Eui Sam Lee, Yong Gyu Choi

**Affiliations:** 1 Department of Materials Science and Engineering, Korea Aerospace University, Gyeonggi, 10540 Republic of Korea; 2 Ultra Precision Optics Research Center, Korea Photonics Technology Institute, Gwangju, 61007 Republic of Korea

## Abstract

A facile procedure for compositional screening of chalcogenide glass (CG) is proposed to manage its infrared transmission edge (*ω*_c_) as well as refractive index dispersion (*ν*) in the long-wavelength infrared (LWIR) range. Both *ω*_c_ and *ν* of CG turn out to be interpretable simply in connection with its chemical composition based on a postulation that CG behaves as a single average harmonic oscillator (SAHO). In this SAHO model, *ω*_c_ is expressed as a function of molar mass and average bond energy, both of which are easily accessible for a given CG composition. Two prototypical CG-forming systems in Ge-Sb-Se and Ge-Sb-S compositions exemplify the empirical compositional dependence of *ω*_c_, which further plays a decisive role in determining *ν*. Following the present approach, a set of highly dispersive CG compositions in the Ge-Sb-S system is newly unveiled together with low-dispersion Ge-Sb-Se glasses. It is then experimentally demonstrated that a doublet lens configuration consisting of convex and concave lenses with low and high *ν* values, respectively, is able to reduce the optical aberrations. This finding presents an opportunity that *ν* can be envisaged just based on the compositional ratio of CG, thus facilitating completion of the LWIR Abbe diagram.

## Introduction

Infrared cameras operating at wavelengths of 8–12 μm, typically referred to as LWIR range, are able to efficiently detect photons emitted from a homoeothermic body whose black body radiation peaks at ~10 μm. In addition to their now existing military applications, demand has been sharply increasing in the field of civilian applications. In particular, efforts to integrate infrared cameras into mobile electronic devices, e.g., smartphone, became a conspicuous trend already^1^. When incorporating the infrared camera module inside a mobile device, its relative size and cost effectiveness should play vital roles together with its thermal-image quality in connection with sensitivity and resolution. Among LWIR-transmitting lens materials, notably, a group of CGs that are inherently mouldable are competitive in terms of processing cost against their counterparts such as single-crystalline Ge and chemical-vapor-deposited poly-crystalline ZnSe^2^. As compositional adjustment of CGs is intrinsically more feasible than the existing crystalline materials, refractive index (*n*) itself and its dispersion can be engineered relatively more straightforwardly. These merits of LWIR-transmitting CGs are able to contribute conspicuously to specific applications where plural lenses with different refractive index dispersions are preferred. It is expected that requirements on performance of such LWIR lenses are rapidly becoming more complicated in order to keep up with technological advancement of LWIR image sensors. In the case of optical glasses for use as lenses working at the visible spectrum, a multitude of glass compositions are readily available and well-categorized with regard to *n* and *ν* (typically expressed as Abbe number)^3^. This exceptionally large variety stems from the compositional flexibility inherent in glass materials, thus allowing extra degrees of freedom in designing lens systems to any desired levels of performance. This reasoning is most likely to be also valid for infrared-transmitting lenses. For example, a doublet configuration of CG lenses with different values of *n* and *ν* would effectively minimize chromatic aberration while simultaneously reducing spherical aberration and other optical aberrations in the LWIR range, just like a doublet (or a group) of oxide glass lenses serves in the visible spectrum.

A majority of optical glasses for lens applications in the visible spectrum are mostly silicate glasses containing various minor elements properly introduced to adjust *n* and *ν*. In view of Clausius-Mossotti relation, *n* of a non-absorbing dielectric material like glass would be specified in connection with both molar volume and polarizability of its constituent atoms. This implies that glass consisting of highly polarizable atoms that are relatively densely packed results in less free volume, and thereby tends to exhibit higher *n* values in general^4–6^. Empirical formulations (and thus derived equations) partly or fully based on the additivity principle have been suggested in an effort to establish compositional dependence of *n* and/or *ν* of optical glasses in the visible spectrum^7^. As far as *n* values concerned, the basic design strategy would also work in the LWIR range. Compared with the case of controlling *n* of optical glasses in the visible spectrum, however, such formulations have been hardly available as for optical glasses in the LWIR spectrum. In addition, we notice that the existing CGs capable of transmitting the LWIR range are all classified as low-dispersion glasses^8–10^. For example, the commercialized Ge-Sb-Se glasses unanimously feature Abbe numbers for the LWIR region, i.e., ${\nu }_{10}=\frac{{n}_{10}-1}{{n}_{8}-{n}_{12}}$, greater than 100^10^, which is defined as using *n* values measured at 8 μm, 10 μm and 12 μm, i.e., *n*_8_, *n*_10_ and *n*_12_, respectively. In this regard, it is further necessary to search for new CGs with higher dispersion in order to configure more compact and better functioning LWIR lens assemblies. Based on these considerations, a part of the present study is aimed to develop CGs that are mouldable, transparent up to at least 12 μm and highly dispersive in refractive index. In this paper, compositional dependence of multiphonon absorption edge (expressed in the form of *ω*_c_) and refractive index dispersion of LWIR-transmitting CGs are mainly discussed with a special emphasis given to high-dispersion CGs. More specifically, ternary or quaternary CG specimens based primarily on Ge-Sb-(Se or S) system were synthesized, and *ω*_c_ values determined from their infrared transmission spectra were examined in an effort to establish a facile and viable means that correlates the *ω*_c_ values with the corresponding CG compositions. Within the framework of the SAHO model, *ω*_c_ turns out to be interrelated with molar mass (*M*) and the average bond energy (*E*_ave_) that can be simply calculated for any given chemical compositions. Taking into consideration Sellmeier equation and the classical Lorentz oscillator model^11^, the infrared-side transmission edge, viz., *ω*_c_ in this study, turns out to be closely related with *ν* over the LWIR range rather than the ultraviolet-side transmission edge by which refractive index dispersion of the conventional silicate-glass-based optical materials is chiefly influenced.

## Materials and Methods

### Glass preparation

The prepared glass compositions out of ternary Ge-Sb-(Se or S) and quaternary Ge-(Ga or In)-Sb-(Se or S) systems were determined in consideration of various properties that need to be paid attention as to the precision glass moulding process, i.e., hardness, glass transition temperature, thermal expansion coefficient, infrared transmittance and refractive index^12^. The number of compositions thus selected was 74 in selenide glasses (Supplementary Figure S1), and 27 in sulfide glasses (Supplementary Figure S2). Bulk glass specimens were synthesized using the conventional melt-quenching technique for synthesis of CGs^13^. Starting materials of Ge, Sb (Advanced Materials), Se (Alfa Aesar), S, Ga, and In (Sigma Aldrich), all in their elemental form, were better than 99.9999% in purity. The starting materials used in this study were in the form of granules or chips of which size varied from 1 to 3 mm. After being weighed inside a nitrogen-filled glove box, each batch set identically to 50 g was sealed inside silica ampoule. Pressure inside the silica ampoule was as low as ~10^−4^ poise, which was achieved by our mechanical vacuum pump. Each silica ampoule was maintained at 1000 °C for 10 h in a rocking furnace to secure homogenization of the melt, which was subsequently water-quenched. Annealing was carried out for 3 h at temperatures lower by 20 °C than glass transition temperatures of the glasses.

### FTIR and Raman measurements

Infrared transmission spectrum was measured using FTIR spectrometer (Spectrum 100, Perkin-Elmer), of which resolution was 4 cm^−1^ in the wavenumber domain, for optically polished disk specimens with thickness of 2 mm. The measurement was repeated 10 times for each glass specimen to obtain an averaged spectrum in the wavenumber range from 400 to 4000 cm^−1^. In order to prepare specimens for Raman measurements, each annealed glass rod was cut and optically polished into a disk with thickness of ~2 mm. FT-Raman spectrometer (FRA 160/S, Bruker) served to obtain Raman spectra at room temperature. The unpolarized 1064 nm emission from an Nd^3+^: YAG laser was used as an excitation source. Output laser power was kept at 30 mW that was low enough to avoid any possible photo-induced effects. Measurements of Stokes Raman scattering intensities were repeated 500 times for each glass specimen in the wavenumber range from 50 to 600 cm^−1^, which were then averaged out to result in its Raman spectrum. Resolution of this FT-Raman spectrometer was estimated to be 8 cm^−1^. As-measured Raman spectra were reduced to compensate the temperature effect^14^. The reduced Raman spectrum thus obtained would be an indicative for the approximate vibrational density of state. The reduced Raman spectra were deconvoluted into distinct Gaussian sub-peaks via least-squares fitting. Then, five Gaussian sub-peaks were employed to calculate *ω*_ave_ for selenide glasses, whereas seven Gaussian sub-peaks for sulfide glasses (see Supplementary Figure S3).

### Refractive index measurement

Refractive index of some representative glasses was measured over a range of wavelengths from 3 μm to 12 μm using the High Precision Automatic Spectrometer-Goniometer (Model 22880, Trioptics) through the minimum deviation method. To secure precision in the refractive index measurement, a fairly large glass ingot with diameter of 35 mm was cut and polished into a prism as large as possible. Precise alignment was achieved by projecting each infrared beam onto a camera, which was then adjusted to provide accurate overlap with the reference beam. The absolute accuracy of the instrument was found to be approximately 0.00001 refractive index units. A photomultiplier tube detector was used over the infrared wavelengths desired in this study. In practice, the accuracy of the measurement was limited by the accuracy of a standard ZnSe prism with which calibration was performed.

### Lens assembly fabrication and focal length measurement

Convex or concave lenses were fabricated as designed in our simulation via direct machining for the sake of convenience of processing. An experimental setup was used for the measurement of focal lengths at different LWIR wavelengths. The setup mainly consisted of IR source, narrow-bandpass filter, collimator and detector. Glowing bar served as an infrared light source whose LWIR wavelengths were monochromatized by the interference filter and subsequently collimated into a parallel beam that was directed towards the lens assembly under test. In order to determine focal length of each lens configuration, we used three interference filters at 9.5, 10.5, and 11.0 μm. The parallel LWIR beam from collimator passed through the lens assembly and then reached the detector.

## Results and Discussion

### Compositional dependence of ω_c_ in selenide glasses

**Figure 1:**
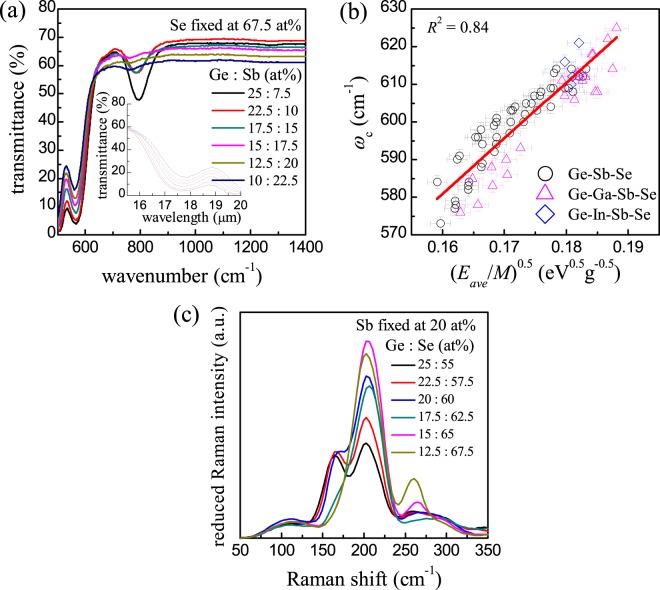
IR and Raman spectra of selenide glasses. (**a**) Representative infrared transmission spectra of a series of Ge-Sb-Se glasses. (**b**) Plot of *ω*_c_ against ${(\frac{{E}_{{\rm{ave}}}}{M})}^{\frac{1}{2}}$ for ternary or quaternary selenide glasses. (**c**) Representative normalized and reduced Raman spectra of a series of Ge-Sb-Se glasses.

As mentioned above, a Ge-rich region inside the glass-forming range of the ternary Ge-Sb-Se system where all of the commercialized glass compositions are included, together with some additional compositions in a Sb-rich region, were made into glass specimens in this study (see Supplementary Figure S1) through the conventional melt-quenching technique for CG preparation. In addition, some quaternary selenide compositions further including Ga or In were prepared to further enhance their thermal and mechanical properties. The selenide glass compositions were selected in consideration of their practicality for use as LWIR lens applications^15^. Taking a look at some representative infrared transmission spectra of the Ge-Sb-Se glasses (Fig. 1a), it is noticed that the *ω*_***c***_ values (determined as the wavenumber at which transmittance falls to half of the baseline transmittance; see Supplementary Figure S4) tend to vary with the changes in relative ratios of the constituent atoms. In this study, vibrational motions of the constituent atoms in CG responsible for appearance of the multiphonon absorption edge is posited as the vibrations of a simple harmonic oscillator (e.g., a diatomic molecule) of which the resonance frequency is represented with force constant (*k*_ave_) and reduced mass (*μ*_***ave***_) as ${(\frac{{{\boldsymbol{k}}}_{{\bf{a}}{\bf{v}}{\bf{e}}}}{{{\boldsymbol{\mu }}}_{{\bf{a}}{\bf{v}}{\bf{e}}}})}^{\frac{1}{2}}$. Numerical values for *k*_ave_ and *μ*_ave_ are not easily computable, so that these are replaced heuristically with more easily accessible parameters, i.e., *E*_ave_ and *M*, respectively, in the scope of our SAHO model. Hence, the quantity ${(\frac{{{\boldsymbol{E}}}_{{\bf{a}}{\bf{v}}{\bf{e}}}}{{\boldsymbol{M}}})}^{\frac{1}{2}}$ is expressed in terms of energy rather than force constant, and *E*_ave_ is presumed conceptually to be equivalent to the potential energy stored in the single average harmonic oscillator. Now, photon energy corresponding to *ω*_c_ in wavenumber is simplified to be ${{\omega }}_{c}\propto {(\frac{{{\boldsymbol{E}}}_{{\bf{a}}{\bf{v}}{\bf{e}}}}{{\boldsymbol{M}}})}^{\frac{1}{2}}$ according to our SAHO model. For any given CG compositions, the values *E*_ave_ and *M* are numerically assessable; in particular, for calculation of *E*_ave_ for each composition of Ge-Sb-Se glass, we employ equations suggested by Tichý and Tichá^16^. Treating the structure of CG as a covalent network, they devised the equations for *E*_ave_ that are expressed in terms of bond energy of atomic pair and coordination number of each constituent atom in CG under consideration (Supplementary Table S1). Figure 1b highlights that a nice linear correlation (*R*^2^ = 0.84) is revealed between *ω*_c_ and ${(\frac{{{\boldsymbol{E}}}_{{\bf{a}}{\bf{v}}{\bf{e}}}}{{\boldsymbol{M}}})}^{\frac{1}{2}}$ values for 74 compositions in total out of ternary Ge-Sb-Se and quaternary Ge-(Ga or In)-Sb-Se glasses. The linear correlation manifests itself again when the infrared transmission edge is determined using a cut-back method that needs measurements of transmission spectra for at least two different thicknesses of glass specimens^17^, and thereby results in absorption coefficients around the infrared transmission edge region (data not shown).

Since *ω*_c_ is supposed to appear as a consequence of multi-processes of the fundamental vibrational transition (*ω*_ave_), we obtain *ω*_c_ = *Pω*_ave_ in which a proportionality factor *P* is introduced. We employ Raman spectra to get an idea as to the correlation between the changes in *ω*_c_ and *ω*_ave_. Presented in Fig. 1c are the representative Raman spectra of Ge-Sb-Se glasses, which reveal that the spectral lineshapes undergo changes in an organized manner upon the compositional variations^18^. Infrared absorption spectra taken in the wavenumber range of the fundamental vibrational excitations would straightforwardly expose the correlation, but too many spectral noises were incorporated in our measurements of such IR spectra. It was thus difficult to utilize the far-infrared spectra for the comparison of *ω*_c_ and *ω*_ave_. The selection rules for Raman-active modes differ from those of IR-active modes^19^, so that these Raman spectra are not necessarily identical with the far-infrared spectra of these glasses with respect to the spectral lineshapes. However, the inherent randomness associated with the atomic arrangements in these glasses would alleviate the selection rules, which allows us to presume that at least the wavenumber range for the major Raman peaks is similar to the range where the fundamental IR absorptions take place^20^. We least-squares fitted the reduced and normalized Raman spectrum with 6 Gaussian sub-peaks (see Supplementary Figure S3 for example)^18^, and the area fraction of each sub-peak was used for calculation of the weighted average given by ${\omega }_{{\rm{ave}}}={\sum }^{}{f}_{i}{\omega }_{i}$ where *f*_*i*_ and *ω*_*i*_ denote the area fraction and the crest position of the *i*-th sub-peak. We then calculated $\frac{{{\rm{\omega }}}_{{\rm{c}}}}{{{\rm{\omega }}}_{{\rm{ave}}}}$ ratios for 40 compositions under consideration to obtain *P* = 2.83 ± 0.06, which means that on average a three-phonon process of *ω*_ave_ makes up *ω*_c_ to be apparent in a typical transmission spectrum of 2-mm-thick selenide glasses. It is worth mentioning that the position of the infrared-side transmission edge, if determined following the procedure employed in this study, varies upon thickness as well as temperature of the glass specimen. In our preliminary experiments devoted to check thickness-dependent changes in spectral features around the IR transmission edge (Supplementary Figure S4), a relatively monotonous but steep drop appeared around the edge region for thicknesses of 1−10 mm, which is considered as typical thickness range for practical infrared transmitting lenses and filters. As such, pinpointing wavenumber for the half-maximum transmittance was done relatively more accurately and expediently when specimens were within this thickness range. The linear correlation between *ω*_c_ and ${(\frac{{E}_{{\rm{ave}}}}{M})}^{\frac{1}{2}}$ was also confirmed when thickness was set to 8 mm (see Supplementary Figure S5). On the other hand, the *ω*_ave_ values plotted as a function of ${(\frac{{E}_{{\rm{ave}}}}{M})}^{\frac{1}{2}}$ values (data not provided) exhibit a poorer correlation as compared with the *ω*_c_ case. This observation leads us to infer that averaging process of the fundamental vibrational modes takes place to reveal the multiphonon absorption edge, so that the *ω*_c_ values becomes more interrelated with ${(\frac{{E}_{{\rm{ave}}}}{M})}^{\frac{1}{2}}$ than the *ω*_ave_ values.

### Compositional dependence of ω_c_ in sulfide glasses

**Figure 2:**
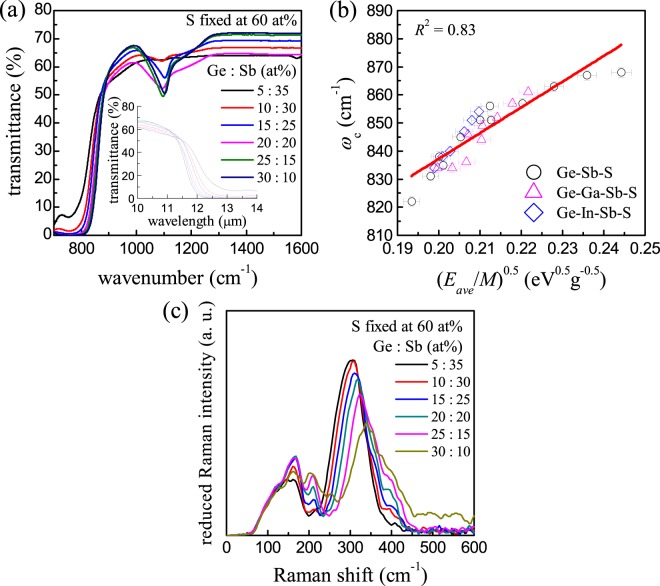
IR and Raman spectra of sulfide glasses. (**a**) Representative infrared transmission spectra of a series of Ge-Sb-S glasses. (**b**) Plot of *ω*_c_ against ${(\frac{{E}_{{\rm{ave}}}}{M})}^{\frac{1}{2}}$ for ternary or quaternary sulfide glasses. (**c**) Representative Raman spectra of a series of Ge-Sb-S glasses.

The empirical relationship derived from the SAHO model has also been verified in the case of sulphur-based Ge-Sb-S glasses with or without a small amount of the fourth constituent such as Ga and In (Supplementary Figure S2). It is worth mentioning that for the purpose of the LWIR lens applications the content of Ge needs to be kept low, i.e., not more than ~15 at%, in order to secure a practically useful transmittance at 12 μm. In our preliminary experiments, thermal and mechanical properties of these glasses turned out to depend most sensitively on the Ge content. In this case, Ga or In was further introduced to improve their thermal and mechanical properties, i.e., glass transition temperature, thermal expansion coefficient and micro-hardness, while maintaining the multi-phonon absorption edge to be much less altered (data not shown). These sulfide glasses feature the multiphonon absorption edge located at around 12 μm which is shorter than that of the Ge-Sb-Se glasses (Fig. 2a). The *ω*_c_ values obtained from the Ge-Sb-S glasses (some glasses additionally containing Ga or In, thus the number amounting to 27 in total) also confirm the linear correlation with the ${(\frac{{{\boldsymbol{E}}}_{{\bf{a}}{\bf{v}}{\bf{e}}}}{{\boldsymbol{M}}})}^{\frac{1}{2}}$ values (*R*^2^ = 0.83) as displayed in Fig. 2b. Here, it is noted that a bimodal variation of *ω*_c_ and ${(\frac{{{\boldsymbol{E}}}_{{\bf{a}}{\bf{v}}{\bf{e}}}}{{\boldsymbol{M}}})}^{\frac{1}{2}}$ with a threshold behavior near ${(\frac{{{\boldsymbol{E}}}_{{\bf{a}}{\bf{v}}{\bf{e}}}}{{\boldsymbol{M}}})}^{\frac{1}{2}}$ = 0.21, i.e., appearance of two straight lines with distinct slopes, might be recognized possibly due to changes in glass structures in a length scale much exceeding the short-range order. If we fitted the plotted data with two straight-line segments, the corresponding *R*^2^ would improve further; however, at this time we reserve such refinements until we gather a lot more experimental data and elaborate *E*_ave_ to reflect the topological aspects of glass structure more precisely. On the other hand, the major peaks in Raman spectra of these sulfide glasses appear to be shifted towards the high-frequency side as compared with the selenide glasses (Fig. 2c), and the same procedure described above as to obtaining *ω*_ave_ values was applied to these sulfide glasses as well. The least-squares fitting was performed on the normalized Raman spectrum with seven Gaussian sub-peaks (Supplementary Figure S3) and the area fraction of each sub-peak was used for obtaining the weighted average. In the case of 20 sulfide glass compositions, *P* was calculated to be 2.93 ± 0.05, thus indicating a three-phonon process of *ω*_ave_ like the situation of selenide glasses.

The atomic arrangements in Ge-Sb-Se and Ge-Sb-S glasses are delineated: The network structures of these two glass families are built with four-fold coordinated Ge atoms and three-fold coordinated Sb atoms in which the chemical preference favoring heteropolar bonds to chalcogen atoms applies to the entire compositions that are able to form glass state through the conventional melt-quenching technique^21–24^. Compositions deviating from the stoichiometric ratio are allowed to form homopolar bonds, e.g., Ge-Ge, Ge-Sb and/or Sb-Sb. In fact, the equations for calculating *E*_ave_ values suggested by Tichý and Tichá is in accordance with this structural model^16^. However, our structural analysis based on Raman spectroscopy indicates that the chemical preference is violated in these glasses; the homopolar bonds appear to exist even in the stoichiometric compositions^18^. As such, the corresponding uncertainty is likely introduced to the calculated *E*_ave_ values, which implies that the degree of the correlation would be enhanced further given that the calculated *E*_ave_ values were more precisely reflecting the actual glass structures in connection with the number of heteropolar bonds relative to that of homopolar bonds. Another source of the uncertainties associated with the linear interrelation between the *ω*_c_ and ${(\frac{{E}_{{\rm{ave}}}}{M})}^{\frac{1}{2}}$ parameters is mentioned: To be consistent with the force constant unit, the parameter *E*_ave_ needs to be divided by a numerical value equivalent to the displacement squared. In our approach, we presume that the vibrational displacement of the SAHO for a given chemical composition is invariable. Because the inter-atomic distance between each atomic pair in these CGs is supposed to change upon differing chemical composition^25–28^, even though insignificant, the corresponding uncertainty would be participated in the calculation of *E*_ave_ values.

### Compositional dependence of ν in chalcogenide glasses

The wavelength-dependent refractive index in the transmission window of optical glasses is well-described through the Sellmeier relation where *A*, *B*_*i*_ and *λ*_*i*_ denote Sellmeier coefficients^29^;

$${n}^{2}(\lambda )=A+{\sum }_{i}^{I}\frac{{B}_{i}{\lambda }^{2}}{{\lambda }^{2}-{\lambda }_{i}^{2}}$$

Even though this Sellmeier equation is often expanded such that *I* > 2 to phenomenologically account for *n* values observed from the visible to the LWIR wavelengths, we rationalize that three-term Sellmeier equation including the constant term *A*, i.e., *I* = 2, is theoretically compatible with the Lorentz oscillator model based on that these CGs possess two absorption resonances in both ends of the optical transmission window, i.e., one present in the UV-side and the other in the IR-side which correspond in this case to *λ*_1_ and *λ*_2_, respectively. The first term incorporating *λ*_1_ affects the dispersion more significantly over the visible wavelengths, whereas the second term with *λ*_2_ does over the IR wavelengths. Specifically, this three-term Sellmeier equation can be considered identical with the Lorentz oscillator model describing a condition of no applied damping force, i.e., the wavelengths in which dielectric materials are optically transparent^30^. As described above, the vibrational motions of constituent atoms in these CGs are assumed in this study to result from a single average harmonic oscillator. The parameter *λ*_2_ then indicates the effective resonance wavelength for the vibrational absorptions of these CGs. It is worth mentioning here that in view of our SAHO model the electric-dipole-induced IR absorptions in the range across the fundamental transitions and their overtones result from vibrational motions of a simple harmonic oscillator, so that the *ω*_ave_ values are set to be equivalent to *λ*_2_ values.

**Figure 3:**
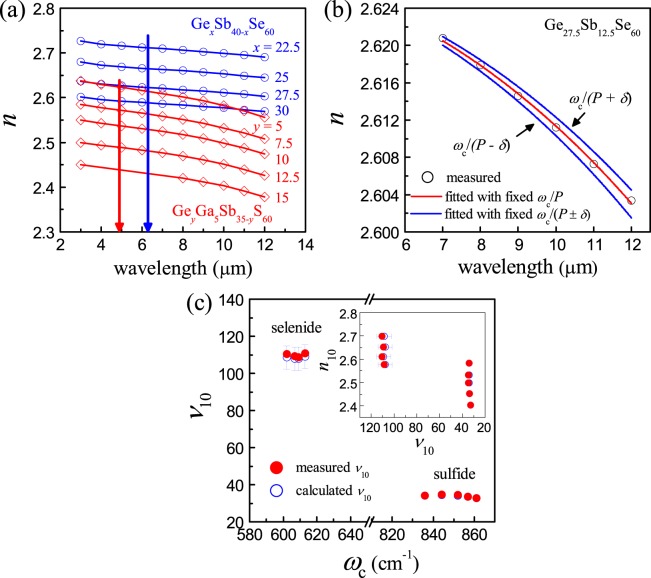
Refractive index dispersion of chalcogenide glasses. (**a**) Measured refractive indexes of the selected Ge-Sb-Se and Ge-Ga-Sb-S glasses. Note that the adjacent data points are connected by a straight line segment for a clear presentation. The dotted arrows indicate the inflection points in wavelength, i.e., ~4.9 μm and ~6.3 μm for sulfide and selenide glasses, respectively. (**b**) Refractive index dispersion curves obtained from the two-term Sellmeier equation least-squares fitted to Ge_27.5_Sb_12.5_Se_60_ (at%) glass. (**c**) Correlations between *ν*_10_ and *ω*_c_ for the selected selenide and sulfide glasses. Inset denotes Abbe diagram in the LWIR region of these CGs. The glass compositions used in this plot are same to those declared in the panel (a) and distinguishable from each other in terms of refractive index.

Taking a look at the measured *n* values of some representative Ge-Sb-Se and Ge-Ga-Sb-S glasses in Fig. 3a, it is noticed that inflection points appear at ~6 μm and ~5 μm, respectively. This would justify that the refractive index dispersion in the spectral range longer than the inflection-point wavelength, viz., the LWIR range, can be described simply with a two-term Sellmeier equation. A more detailed explanation for this rationalization is available in Supplementary Figure S6. On the basis of this observation, the changes of the measured *n* values in the LWIR range are supposedly caused mainly by the vibrational absorption rather than the bandgap absorption. Then, in this situation, the dispersion induced by the bandgap transition is not wavelength-dependent in the LWIR range, so that this electronic contribution is merged into the constant *A*. As for most silicate-glass-based optical materials, the refractive index dispersion chiefly depends on position and magnitude of the UV-side absorption that takes shape in the form of the Tauc edge^31^. Among the multiple models that have been suggested to describe the absorption properties around the Tauc gap, the Tauc-Lorentz model and its derivatives would be capable of explaining the dispersive characteristics of the silicate glasses in the visible spectrum^32,33^. Contrary to this, in the case of CGs, the IR absorption due to vibrational excitations would be more influential to the dispersion properties in the LWIR range. In this study, we allow *λ*_2_ in the two-term Sellmeier equation to be substituted with the reciprocal of $\frac{{\omega }_{c}}{P}$ in consideration of the difference in the units between wavelength and wavenumber, which is thus expressed as follows;

$${n}^{2}({\rm{\lambda }})=A+\frac{{B}_{2}{{\rm{\lambda }}}^{2}}{{{\rm{\lambda }}}^{2}-{(\frac{{\omega }_{c}}{P})}^{-2}}$$

We observe that the measured *n* values are fitted nicely across the LWIR range with the above two-term Sellmeier equation in which we set only the two Sellmeier coefficients, i.e., *A* and *B*_2_, to be determined from the least-squares curve fitting. (Fig. 3b). This implies that the resonance absorption wavelength *λ*_2_ can be effectively deduced from *ω*_c_, thus reasonably accounting for the refractive index dispersion in the LWIR spectrum. In this case, when we take into consideration the standard deviation involved in the proportionality factor, i.e., *P* ± *δ*, in our curve fitting, the resulting dispersion curves become somewhat deviated as presented also in Fig. 3b, but discrepancy tantamount to less than 0.01 appears between the measured *n* values and the dispersion curves.

For the sake of convenience, the Abbe number *ν*_10_ is introduced to compare dispersions of the selenide and sulfide glasses. Displayed in the inset of Fig. 3c is the Abbe diagram in the LWIR spectrum in which the measured values for *n*_10_ and *ν*_10_ of some representative compositions of the present CGs are plotted together with the range of *ν*_10_ values obtained from our method. Selenide glasses are categorized as low-dispersion glasses, as expected, whereas sulfide glasses as high-dispersion glasses. In an effort to disclose the interrelation between *ν*_10_ and *ω*_c_ for these glasses, we plot *ν*_10_ and *ω*_c_ simultaneously in Fig. 3c, which reveals a rough but clear interdependence between these two quantities. Based on our SAHO model, wavelength-dependent refractive index in the LWIR spectrum would be directly deduced from chemical composition of CGs. It is worth mentioning that all of the sulfide glasses displayed in Fig. 3c show *ν*_10_ values less than 40, thus being classified well as high-dispersion CGs.

These sulfide glasses are classified as a group of high-dispersion CGs newly found in this study, and thus other CG-forming compositional systems such as mixed-chalcogen compositions, i.e., glasses containing both S and Se, would also be highly dispersive in the LWIR region.

### LWIR lens assembly consisting of chalcogenide glasses

**Figure 4:**
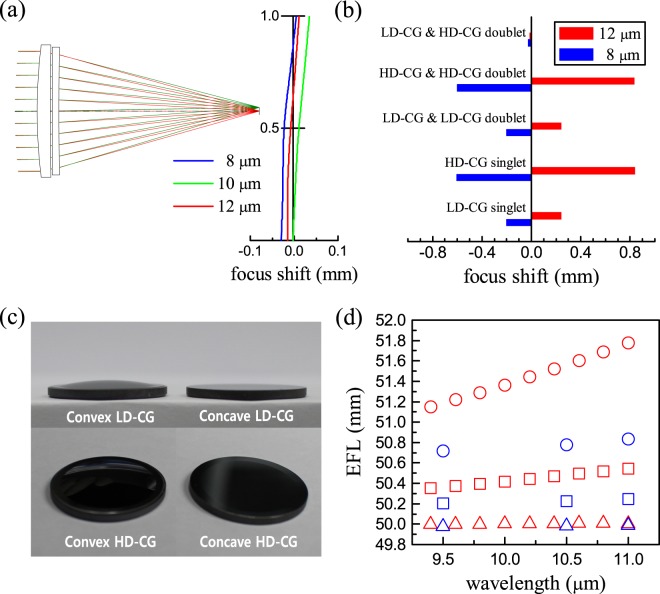
LWIR lens assembly made of chalcogenide glasses. (**a**) Graphical expression for the doublet configuration composed of convex LD-CG and concave HD-CG lenses, and calculated focus shifts at wavelengths of 8, 10 and 12 μm with respect to the focal length initially set to 50 mm at 10 μm at the center position. (**b**) Calculated focus shifts at 8 and 12 μm from the focal length at 10 μm for the five different lens configurations. (**c**) Photographs of the LD-CG and HD-CG lenses in the form of either convex or concave geometrical shape fabricated in this study. (**d**) Effective focal lengths (EFLs) over the LWIR wavelengths calculated (color in red) or measured (color in blue) for three different doublet configurations: The circular, square and triangular symbols indicate the cases for the convex HD-CG & concave HD-CG, the convex LD-CG & concave LD-CG, and the convex LD-CG & concave HD-CG, respectively. Note that uncertainties involved in the measured data are smaller than the size of each symbol.

In an effort to corroborate the positive attributes of a lens assembly combining the high- and low-dispersion CGs simultaneously, two representative CG compositions were selected mainly in view of *n*_10_ and *ν*_10_ as well as processability as to lens formation: Ge_27.5_Sb_12.5_Se_60_ and Ge_5_Ga_5_Sb_30_S_60_ (at.%) as the low-dispersion CG (denoted LD-CG hereafter) and the high-dispersion CG (HD-CG), respectively. Note that *n*_10_ and *ν*_10_ values of 2.6112 and 110.81 were respectively obtained from our measurements for LD-CG, whereas 2.5822 and 34.34 for HD-CG. These two CGs were utilized in our numerical simulations accounting for singlet or doublet lenses of which diameter and focal length were identically set to 20 mm and 50 mm at wavelength of 10 μm, respectively. Specifically, two convex singlets and three doublets consisting of a pair of convex and concave lenses were taken into consideration for the present LD-CG and HD-CG, as shown in Supplementary Figure S7. Presence of chromatic and spherical aberrations involved in a lens assembly manifests itself as a shift of the focal length over wavelength. Thus, those focal lengths at three different wavelengths, i.e., 8, 10 and 12 μm, were calculated in order to compare the optical aberrations of those lens configurations under consideration in this study (see Fig. 4a and Supplementary Figure S7). For a clarity of comparison for the optical aberrations, shifts of the focal lengths at wavelengths of 8 μm and 12 μm with respect to the focal length at 10 μm are displayed in Fig. 4b for the five different lens configurations, i.e., convex LD-CG & concave HD-CG doublet, convex HD-CG & concave HD-CG doublet, convex LD-CG & concave LD-CG doublet, convex HD-CG singlet, and convex LD-CG singlet. It is noticed that for the two singlet configurations the LD-CG convex singlet exhibits focus shifts much reduced for both wavelengths as compared with the HD-CG convex singlet, thus providing much reduced optical aberrations. In addition, for these specific surface curvatures of the convex and concave lenses, the doublet configurations consisting of either LD-CG only or HD-CG only result in a marginal improvement of the optical aberrations. On the contrary, however, the corresponding focus shifts are markedly reduced by approximately one order of magnitude in the case of the doublet configuration where the convex LD-CG lens and the concave HD-CG lens are combined together.

In order to experimentally justify the advantages of combining high- and low-dispersion chalcogenide glasses simultaneously, the LD-CG and HD-CG were fabricated into convex or concave lenses following the geometrical dimension and optical power outlined above, as displayed in Fig. 4c. After optically aligning the convex-concave doublet configurations out of the LD-CG and HD-CG lenses, the effective focal length (EFL) of each doublet configuration was measured and then compared with the calculated EFL for the corresponding doublet configuration. As graphically demonstrated in Fig. 4d, the doublet lens module out of HD-CG lenses only shows EFL difference of 0.12 mm between 9.5 μm and 11.0 μm. This EFL difference becomes 0.04 mm for the doublet assembled only with LD-CG lenses. Notably, EFL of 0.01 mm is observed for the doublet lens module composed of the convex LD-CG and concave HD-CG lenses, thus confirming the benefits of the simultaneous use of high- and low-dispersion chalcogenide glasses. It is also noticed in Fig. 4d that even though there are mismatches between the experimentally measured EFL values and the calculated ones, the doublet configuration employing the convex LD-CG and concave HD-CG lenses reveals the calculated EFL values, which are significantly decreased over the LWIR range as compared with the other two doublet configurations.

## Conclusions

Two prototypical CGs mostly out of ternary Ge-Sb-Se and Ge-Sb-S compositions have been employed in an effort to elucidate the compositional dependence of *ω*_c_ and *ν* in the LWIR spectral range. In this study, CG is presumed to act as a single average harmonic oscillator, and thereby the *ω*_c_ values measured from the selenide and sulfide glasses are discovered to be linearly proportional to the corresponding ${(\frac{{E}_{{\rm{ave}}}}{M})}^{\frac{1}{2}}$ values. This finding implies that the infrared absorption behaviors of CGs, the *ω*_c_ values in particular, can be predicted using their chemical compositions via average bond energy and molar mass. In addition, the weighted average of Raman sub-peaks in these CG families appears to be closely related with the *ω*_c_ value: The $\frac{{{\rm{\omega }}}_{{\rm{c}}}}{{{\rm{\omega }}}_{{\rm{ave}}}}$ ratio is verified, in the case of glass thickness of 2 mm, to be 2.83 ± 0.06 for selenide glasses, and 2.93 ± 0.05 for sulfide glasses. This result indicates that a three-phonon process of the averaged vibration mode renders the infrared-side absorption edge appear when CGs are practically thick. The two-term Sellmeier equation adequately describes the dispersion behaviors of CGs in the LWIR range, and the *n* values measured over the LWIR wavelengths turn out to be well fitted with the two-term Sellmeier equation incorporating the *ω*_c_ value for a given CG. A set of highly dispersive Ge-Ga-Sb-S glasses is newly unveiled based on the present approach. A doublet lens assembly consisting of CGs with high and low *ν* values was demonstrated not only to significantly reduce the optical aberrations but also to downsize the lens assembly. In summary, Abbe numbers in the LWIR range are facilely deduced from compositional ratios of CG in view of the SAHO model and the experimentally determined $\frac{{{\rm{\omega }}}_{{\rm{c}}}}{{{\rm{\omega }}}_{{\rm{ave}}}}$ ratio. It would be interesting to check whether or not the SAHO model is expanded to other glasses, e.g., oxide glasses.

## Electronic supplementary material

Supplementary information
