# Supplementary material for: Unravelling interrelations between chemical composition and refractive index dispersion of infrared-transmitting chalcogenide glasses
Source: Sci Rep. 2018 Oct 19;8:15482. doi: 10.1038/s41598-018-33824-x (PMC6195534; doi:10.1038/s41598-018-33824-x)
Supplement: Supplementary file 1 — Supplementary information [file 41598_2018_33824_MOESM1_ESM.docx]

Supplementary Information

Unravelling interrelations between chemical composition and refractive index dispersion of infrared-transmitting chalcogenide glasses

Jun Ho Lee^1,+^, Ju Hyeon Choi^2,+^, Jeong Han Yi^1^, Woo Hyung Lee^1^, Eui Sam Lee^2^, and Yong Gyu Choi^1,*^

^1^Department of Materials Science and Engineering, Korea Aerospace University, Gyeonggi 10540, Republic of Korea

^2^Ultra Precision Optics Research Center, Korea Photonics Technology Institute, Gwangju 61007, Republic of Korea

^*^ygchoi@kau.ac.kr

^+^these authors contributed equally to this work

**Selenide Glass Compositions**

The ternary selenide glass compositions prepared in this study are shown in Figure S1. The majority of the prepared glasses are all inside the glass-forming region,^1^ and belong to a Ge-rich region in consideration of practicality concerning the LWIR lens applications. Together with the ternary selenide glasses, some quaternary glasses further incorporating Ga or In were also prepared. In the case of quaternary selenide glasses, compositions in which the fourth constituent replaces Sb are; Ge_30_Sb_10-_*_x_*Se_60_Ga*_x_* (*x* = 1, 3, 5 or 8 at%), Ge_30_Sb_10-_*_x_*Se_60_In*_x_* (*x* = 1 or 3 at%), Ge_27.5_Sb_12.5-_*_x_*Se_60_Ga*_x_* (*x* = 1, 3, 5, 8 or 10 at%), Ge_27.5_Sb_12.5-_*_x_*Se_60_In*_x_* (*x* = 3 or 5 at%), Ge_12.5_Sb_15_Se_62.5_G_10_ and Ge_10_Sb_15_Se_65_G_10_. When the fourth constituent replaces Ge, the compositions are; Ge_25-_*_x_*Sb_10_Se_65_Ga*_x_* (*x* = 5, 10 or 15 at%), Ge_27.5-_*_x_*Sb_7.5_Se_65_Ga*_x_* (*x* = 5, 10 or 15 at%), Ge_25-_*_x_*Sb_7.5_Se_67.5_Ga*_x_* (*x* = 5, 10 or 15 at%) and Ge_27.5-_*_x_*Sb_12.5_Se_60_Ga*_x_* (*x* = 10 or 15 at%).

**
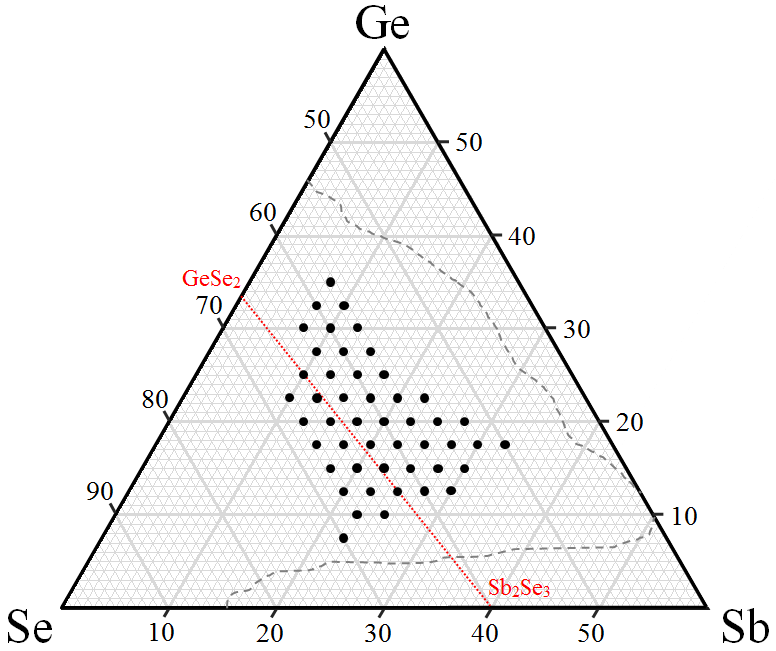
**

**Figure S1** Compositions of ternary Ge-Sb-Se glasses (at%) selected in this study. Note that the dotted straight line denotes the stoichiometric tie-line so that the majority of actual glass specimens prepared belongs to the Ge-rich region.

**Sulfide Glass Compositions**

Shown in Figure S2 are the ternary sulfide glass compositions prepared in this study. Compositions containing relatively small amount of Ge were intentionally chosen considering practicality associated with the LWIR lens applications. Quaternary sulfide glasses incorporating Ga were also prepared in consideration of glass stability. The quaternary sulfide glasses have compositions of Ge_5_Sb_35-_*_x_*S_60_Ga*_x_* (*x* = 1, 3 or 5 at%), Ge_5_Sb_35-_*_x_*S_60_In*_x_* (*x* = 1, 3 or 5 at%), Ge_10_Sb_30-_*_x_*S_60_Ga*_x_* (*x* = 1, 3 or 5 at%), Ge_10_Sb_30-_*_x_*S_60_In*_x_* (*x* = 1, 3 or 5 at%) and Ge*_x_*Sb_35_S_60_Ga_5_ (*x* = 7.5, 12.5 or 15%).


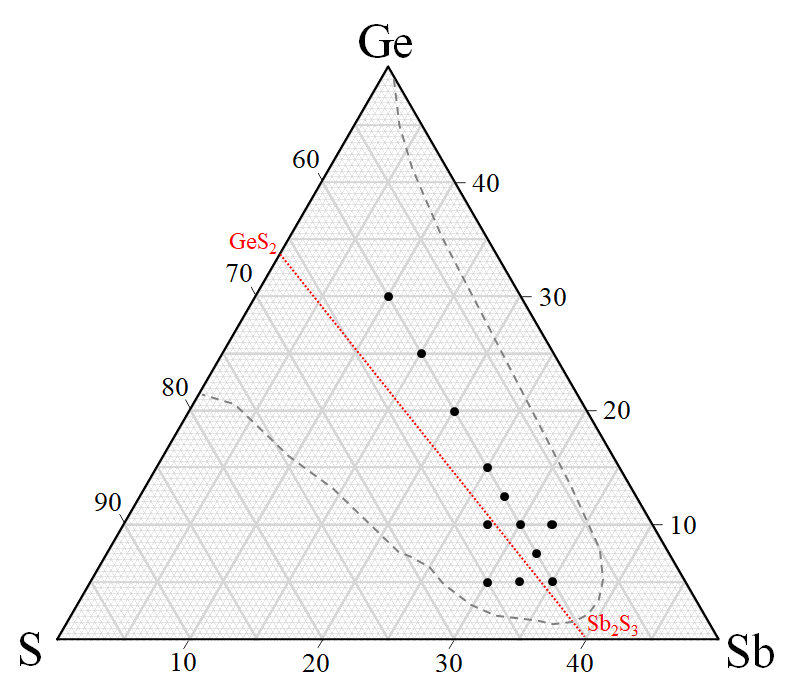


**Figure S2** Compositions of ternary Ge-Sb-S glasses (at%) selected in this study. Note that the dotted straight line denotes the stoichiometric tie-line. The glass-forming region is marked with the curved dotted line.^1^

**Raman Spectra of Selenide and Sulfide Glasses**

The reduced Raman spectrum of selenide glasses was deconvoluted into 6 Gaussian sub-peaks including a sub-peak indicating the constantly appearing instrumental feature (Figure S3a), taking into consideration structural units formed in these glasses.^2-7^ Meanwhile, according to the structural models based on Raman analyses of sulfide glasses,^8-16^ each reduced Raman spectrum was deconvoluted into 8 Gaussian sub-peaks including a sub-peak indicating the constantly appearing instrumental feature (Figure S3b).

**Figure S3** Representative examples showing the least-squares fitted Raman sub-peaks for a) selenide and b) sulfide glasses. Note that the sub-peak located at ~120 cm^-1^ appeared constantly as an instrumental feature for all of the measurements, so that it was neglected in calculating the $\omega_{\mathrm{ave}}$ values.

**Thickness-Dependent Changes of** $\boldsymbol{\omega}_{\mathbf{c}}$ **in Selenide Glasses**

Since the parameter $\omega_{c}$ is determined from infrared transmission spectrum of bulk CGs, it is considered as an extensive property depending thickness and temperature. In an effort to check its thickness dependence, we have varied thickness of selenide glass with a representative composition from 0.3 mm to 55 mm (Figure S4a), and monitored the corresponding changes of $\omega_{c}$ determined as wavenumber at which transmittance falls to 50% of the baseline transmittance (Figure S4b). In the thickness range for typical IR lenses, i.e., 1−10 mm, the $\omega_{c}$ values appear linearly proportional to the glass thickness (Figure S4c).

**Figure S4** a) Thickness-dependent infrared transmission spectra of Ge_25_Sb_15_Se_60_ (at.%) glass. b) Normalized infrared transmission spectra where the position of $\omega_{c}$ is marked, if available, with the circular dot. c) The $\omega_{c}$ values plotted as a function of thickness for some representative selenide glass compositions.

**Bond Energy of Atomic Pairs Formed in Selenide and Sulfide Glasses**

The average bond energy ($E_{\mathrm{ave}}$), which corresponds to enthalpy of atomization, was calculated using the equations given by Tichý and Tichá.^17^ Bond energy of each atomic pair used for the calculations is listed Table S1. Note that the coordination number of Ga and In in quaternary Ge-(Ga or In)-Sb-(S or Se) glasses was set to 4 according to previous strucutral analysis results.

**Table S1** Bond energy of each atomic pair formed in selenide and sulfide glasses.

| Atomic pair | Bond energy  [eV] | Atomic pair | Bond  energy  [eV] |
| --- | --- | --- | --- |
| Ge-Ge | 1.63 | Ga-Ga | 1.48 |
| Sb-Sb | 1.31 | Ga-Ge | 1.60 |
| Ge-Sb | 1.48 | Ga-Sb | 1.46 |
| Se-Se | 1.90 | Ga-Se | 2.39 |
| Ge-Se | 2.40 | Ga-S | 2.57 |
| Sb-Se | 1.86 | In-In | 1.30 |
| S-S | 2.20 | In-Ge | 1.47 |
| Ge-S | 2.40 | In-Sb | 1.39 |
| Sb-S | 2.06 | In-Se | 2.28 |
|  |  | In-S | 2.52 |

**Interrelations between the** $\boldsymbol{\omega}_{\mathbf{c}}$ **and** $\left( \frac{\boldsymbol{E}_{\mathbf{ave}}}{\boldsymbol{M}} \right)^{\frac{\mathbf{1}}{\mathbf{2}}}$ **Parameters**

As stated above, the parameter $\omega_{c}$ is dependent on the thickness of CGs, so the differing $\omega_{c}$ values were plotted as a function of the parameter $\left( \frac{E_{\mathrm{ave}}}{M} \right)^{\frac{1}{2}}$. Shown in Figure S5 are the $\omega_{c}$ values obtained from two thicknesses, i.e., 8 mm and 4 mm for selenide and sulfide glasses, respectively, still exhibit the linear correlations with the $\left( \frac{E_{\mathrm{ave}}}{M} \right)^{\frac{1}{2}}$ values. Note, however, that the 8-mm thickness data were measured using two different FTIR instruments, which is mainly responsible for the relatively loose correlation.

**Figure S5** The $\omega_{c}$ values obtained for two different thicknesses plotted as a function of the corresponding $\left( \frac{E_{\mathrm{ave}}}{M} \right)^{\frac{1}{2}}$ values in the case of a) selenide and b) sulfide glasses.

**Sellmeier Equation of LWIR-Transmitting Chalcogenide Glass**

Sellmeier equation of a commercialized selenide glass is given below;^18^

$$n^{2}=3.46713+\frac{2.8365\lambda^{2}}{\lambda^{2}-0.13451}+\frac{0.95749\lambda^{2}}{\lambda^{2}-1395.5}$$

We checked how significant each term in the Sellmeier equation affects the refractive index dispersion across the LWIR region. As displayed in Figure S6, the third term is critically governing the LWIR dispersion, which implies that the second term can be taken as a constant, so being merged with the first term.

**Figure S6** Effect of each term in the Sellmeier equation of a commercialized selenide glass.

**Simulation of Chalcogenide Glass Lenses**

As displayed in Figure S7, five different lens configurations were taken into consideration for our simulation: two concave singlet configurations out of LD-CG and HD-CG, respectively, and three convex-concave doublet configurations consisting of either LD-CG only, HD-CG only or LD-CG & HD-CG. Diameter and focal length were set to be 20 mm and 50 mm at 10 μm for each lens. The lens designs and corresponding numerical calculations were performed using optical design software CODE V.

**Figure S7** Graphical expression for each lens configuration and the corresponding focus shifts at 8, 10 and 12 μm: a) LD-CG convex singlet, b) HD-CG convex singlet, c) doublet composed of LD-CGs, d) doublet composed of HD-CGs, and e) doublet composed of LD-CG & HD-CG.

**References**

1. Popescu, M. A. *Non-crystalline Chalcogenide* (Kluwer Academic Publishers, Dordrecht, Netherlands, 2002).
2. Ivanova, Z. G., Pamukchieva, V. & Vlcek, M. On the structural phase trasformations in Ge*_x_*Sb_40-_*_x_*Se_60_ glasses. *J. Non-Cryst. Solids* 293-295, 580-585 (2001).
3. Boolchand, P., Feng, X. & Bresser, W.J. Rigidity transitions in binary Ge-Se glasses and the intermediate phase. *J. Non-Cryst. Solids* 293-295, 348-356 (2001).
4. Olivier, M., Tchahame, J.C., Nemec, P., Chauvet, M., Besse, V., Cassagne, C., Boudebs, G., Renversez, G., Boidin, R., Baudet, E. & Nazabal, V. Structure, nonlinear properties, and photosensitivity of (GeSe_2_)_100-_*_x_*(Sb_2_Se_3_)*_x_* glasses. *Opt. Mater. Express* 4, 525-540 (2014).
5. Gan, Y. -L., Wang, L., Su, X. -Q., Xu, S. -W., Shen, X. & Wang, R. -P. Thermal conductivity of Ge*_x_*Sb(As)*_y_*Se_100-_*_x_*_-_*_y_* glasses measured by Raman scattering spectra. *J. Raman Spectrosc.* 45, 377-382 (2014).
6. Wei, W. -H., Fang, L., Shen, X. & Wang, R. -P. Transition threshold in Ge*_x_*Sb_10_Se_90-_*_x_* glasses. *J. Appl. Phys*. 115, 113510 (2014).
7. Wei, W. -H., Wang, R. -P., Shen, X., Fang, L. & -Davies, B. L. Correlation between structural and physical properties in Ge-Sb-Se glasses. *J. Phys. Chem. C* 117, 16571-16576 (2013).
8. Li, Z., Lin, C., Qu, G., Nie, Q., Xu,T. & Dai, S. Phase separation in nonstoichiometry Ge-Sb-S chalcogenide glasses. *J. Am. Ceram. Soc.* 97, 793-797 (2014).
9. Koudelka, L. & Pisarcik, M. Raman spectra of Ge-Sb-S system glasses in the S-rich region. *J. Non-Cryst. Solids* 41, 171-178 (1980).
10. Iliopoulos, K., Hatzikyriakos, G., Couris, S., Ren, J., Wagner, T., Frumar, M., Kyriazis, F. & Yannopoulos, S.N. GeS_2_-Ga_2_Se_3_-AgI glasses with high non-linear optical properties. *International Conference on Transparent Optical Networks* 2, 1-4 (2011).
11. Guo, H., Chen, H., Hou, C., Lin, A., Zhu, Y., Lu, S., Gu, S., Lu, M. & Peng, B. The third-order optical nonlinearities of Ge-Ga-Sb(In)-S chalcogenide glasses. *Mater. Res. Bull.* 46, 765-770 (2011).
12. Kohoutek, T., Yan, X., Shiosaka, T. W., Yannopoulos, S. N., Chrissanthopoulos, A., Suzuki, T. & Ohishi, Y. Enhanced Raman gain of Ge-Ga-Sb-S chalcogenide glass for highly nonlinear microstructured optical fibers. *J. Opt. Soc. Am. B* 28, 2284-2290 (2011).
13. Huang, C. C., Wu, C. C., Knight, K. & Hewak, D. W. Optical properteis of CVD grown amorphous Ge-Sb-S thin films. *J. Non-Cryst. Solids* 356, 281-285 (2010).
14. Frumarova, B., Nemec, P., Frumar, M., Oswald, J. & Vlcek, M. Synthesis and optical properties of the Ge-Sb-S:PrCl_3_ glass system. *J. Non-Cryst. Solids* 256-257, 266-270 (1999).
15. Musgraves, J. D., Carlie, N., Hu, J., Petit, L., Agarwal, A., Kimerling, L. C. & Richardson, K. A. Conparison of the optical thermal and structural properties of Ge-Sb-S thin films deposited using thermal evaporation and pulsed laser deposition techniques. *Acta Mater.* 59, 5032-5039 (2011).
16. Hu, J., Tarasov, V., Carlie, N., Petit, L., Agarwal, A., Richardson, K. & Kimerling, L. Exploration of waveguide favrication from thermally evaporated Ge-Sb-S glass films. *Opt. Mater.* 30, 1560-1566 (2008).
17. Tichý, L. & Tichá, H. Covalent bond approach to the glass-transition temperature of chalcogenide glasses. *J. Non-Cryst. Solids* 189, 141-146 (1995).
18. Schott AG, *IRG22 product information*, http://www.schott.com. Accessed March, 2015.
